# Guideline Recommendations for Oral Care After Acquired Brain Injury: Protocol for a Systematic Review

**DOI:** 10.2196/17249

**Published:** 2020-07-01

**Authors:** Nalia Gurgel-Juarez, Marie-France Perrier, Tammy Hoffmann, Natasha Lannin, Laura Jolliffe, Rachel Lee, Lucie Brosseau, Heather Flowers

**Affiliations:** 1 School of Rehabilitation Sciences Faculty of Health Sciences University of Ottawa Ottawa, ON Canada; 2 Centre for Research in Evidence-Based Practice Faculty of Health Sciences and Medicine Bond University Gold Coast Australia; 3 Occupational Therapy Department Alfred Health Hospital Melbourne Australia; 4 School of Allied Health Faculty of Science, Health and Engineering La Trobe University Melbourne Australia; 5 Department of Neuroscience Central Clinical School Monash University Melbourne Australia; 6 The Ottawa Hospital Research Institute Ottawa, ON Canada; 7 Canadian Partnership for Stroke Recovery Ottawa, ON Canada; 8 Institut du Savoir Montfort Ottawa, ON Canada; 9 Toronto General Hospital University Health Network Toronto, ON Canada

**Keywords:** practice guideline, evidence-based recommendations, brain injuries, oral health, oral hygiene, systematic review

## Abstract

**Background:**

Oral care is important to prevent buccal and systemic infections after an acquired brain injury (ABI). Despite recent advancements in the development of ABI clinical practice guidelines, recommendations for specific clinical processes and actions to attain adequate oral care often lack information.

**Objective:**

This systematic review will (1) identify relevant ABI clinical practice guidelines and (2) appraise the oral care recommendations existing in the selected guidelines.

**Methods:**

A search strategy was developed based on a recent systematic review of clinical practice guidelines for ABI. The protocol includes a search of MEDLINE, EMBASE, and DynaMed Plus databases, as well as organizational and best-practice websites and reference lists of accepted guidelines. Search terms will include medical subject headings and user-defined terms. Guideline appraisal will involve the Appraisal of Guidelines for Research and Evaluation II ratings, followed by a descriptive synopsis for oral care recommendations according to the National Health and Medical Research Council evidence levels.

**Results:**

This project started in April 2019, when we developed the search strategy. The preliminary search of databases and websites yielded 863 and 787 citations, respectively, for a total of 1650 citations. Data collection will start in August 2020 and we expect to begin disseminating the results in May 2021.

**Conclusions:**

Nursing staff may not have detailed recommendations on how to provide oral care for neurologically impaired patients. The findings of this review will explore the evidence for oral care in existing guidelines and improve outcomes for patients with ABI. We expect to provide adequate orientations to clinicians, inform policy and guidelines for best practices, and contribute to future directions for research in the ABI realm.

**International Registered Report Identifier (IRRID):**

PRR1-10.2196/17249

## Introduction

Poor oral health may lead to colonization by various microorganisms, causing buccal and respiratory infections [1-5]. Pneumonia, a life-threatening respiratory infection, is a common cause of death after vascular acquired brain injury (ABI) [6] and can be prevented through effective oral hygiene [7-9]. Good oral health is important to preserve one’s overall health and well-being [10]. To maintain good oral health, evidence-based oral care practices are essential.

Managing oral care after an acquired brain injury is challenging. The damage that occurs to the brain due to traumatic or nontraumatic causes can lead to physical, behavioral, emotional, and cognitive impairments [11]. For instance, stroke—a nontraumatic, common cause of ABI—leaves one-third of survivors with long-term impairments [12], which may prevent them from managing their oral care. As a result, many are partially or totally reliant on caregivers to maintain their oral hygiene [13]. Nursing staff and family members are usually the ones who provide oral hygiene to care-dependent neurologically impaired survivors [14].

However, clinical barriers may restrict the delivery of high-quality oral care. Insufficient oral care knowledge and training are reported as some of the obstacles for nursing staff to perform adequate care [15,16]. Consequently, some professionals feel unprepared [17,18] and prioritize other care activities in the neurosciences context [19,20]. In addition, due to an overwhelming work routine in hospitals and long-term care facilities [21-23], nurses sometimes delegate oral care tasks to the least qualified members of the care team [18].

There is little direction from evidence-based literature regarding oral care provision [18,24,25], which results in great variability of practice across the care continuum [26]. Notwithstanding, there have been recent advancements in the development of clinical practice guidelines for ABI, yet they lack specific clinical instructions and processes for adequate and safe oral care provision [27-29]. A comprehensive understanding of existing oral care recommendations in ABI guidelines is badly needed.

The objectives of our proposed systematic review are to (1) update and extend the appraisal of ABI guidelines conducted in a recent systematic review [30], and (2) review and appraise existing oral care recommendations in included guidelines. Since guidelines should ideally be updated every 2 to 3 years [31] and the recent systematic review included ABI guidelines up to 2017 [30], we expect to identify new documents from our updated search. The reasons for extending the search are to help identify literature on oral health across the continuum of care (now including acute settings) and across ABI severity ranges (now including mild ABI). All oral care recommendations will be extracted from the included guidelines and rated according to their levels of evidence. This review will therefore provide a synthesis of best practices for health care professionals, which we anticipate will inform guidelines and practice standards. In addition, our results will provide insight and direction for future research.

## Methods

### Scope of the Protocol

This protocol is adapted from a recent systematic review of clinical practice guidelines for ABI [30]. Our review seeks to synthesize guidelines relevant to a broader range of settings and severities beyond this previous review. Therefore, our search strategy was expanded to include additional relevant information for the acute setting and for mild ABI. Protocol amendments, if necessary, will be incorporated in future publications, with details such as date, description, and rationale of each amendment.

### Eligibility Criteria

Eligible guidelines will include acute and rehabilitation (inpatient and community rehabilitation) settings and pertain to mild to severe ABI. For the purpose of the current protocol, ABI refers to any damage to the brain that occurs after birth and is not related to a congenital or a degenerative disease [11]. Therefore, causes will include traumatic injury, seizures, tumors, infectious diseases, events in which the brain has been deprived of oxygen, and toxic exposure, such as substance abuse [11].

We will search guidelines published from January 1, 2006, onwards, following the methods of the recent systematic review [30]. Eligible guidelines will be guidelines published in English, particularly those produced under the support of (1) a health professional association or society, (2) a public or private organization, (3) a health care organization or plan, or (4) a government agency [32]. Further, included clinical practice guidelines must contain recommendations, strategies, or information to orient health care professional decisions. Eligible guidelines must have more than 1 component of post-ABI recommendations pertaining to adult patients [27]. In case of incomplete information, we will contact authors of particular guidelines for elaboration or clarification.

### Information Sources and Search Strategy

The protocol combines multiple information sources, including databases as well as organizations and professional websites. Our search will involve MEDLINE, EMBASE, and DynaMed Plus databases. We have developed the search terms for MEDLINE, as seen in Textbox 1, using medical subject headings and user-defined terms. We will apply corresponding terms to the other 2 databases. Subsequently, we will search for organizational and best-practice websites for additional published guidelines.

**MEDLINE search strategy.**

1. exp Craniocerebral Trauma/

2. exp Stroke/

3. exp Anoxia/

4. exp Hypoxia, Brain/

5. ((brain or head or intracran* or cerebr* or cerebellar or brainstem or vertebrobasilar) adj3 (injur* or infarc* or isch?em* or thrombo* or apoplexy or emboli* or h?emorrhag* or h?ematoma* or aneursym* or anoxi* or hypoxi*)).ab,ti.

6. (encephaliti* or mening*).ab,ti.

7. 1 or 2 or 3 or 4 or 5 or 6

8. rehabilitation.fs.

9. exp Rehabilitation/

10. exp Rehabilitation Centers/

11. rehabilitat*.ab,ti.

12. subacute care/

13. short term.ab,ti.

14. acute.ab,ti.

15. (hyperacute or hyper-acute).ab,ti.

16. ((short or urgent or emergency or acute) adj3 (term or care or stay)).ab,ti.

17. ((subacute or sub-acute) adj3 (care or stay)).ab,ti.

18. ((subacute or sub-acute or hyperacute or hyper-acute) adj3 (care or stay)).ab,ti.

19. 8 or 9 or 10 or 11 or 12 or 13 or 14 or 15 or 16 or 17 or 18

20. exp guideline/

21. Guideline$.ti.

22. (guideline or practice guideline).pt.

23. 20 or 21 or 22

24. 7 and 19 and 23

### Protocol Guidelines

The design of this systematic review follows the Preferred Reporting Items for Systematic Review and Meta-Analysis (PRISMA) [33] and PRISMA-Protocols [34,35] guidelines.

### Study Records

#### Data Management and Collection

Two independent reviewers (NG-J and M-FP) will use the web-based software Covidence (Veritas Health Innovation Ltd) [36] and the RevMan (Cochrane) software [37] to support and streamline the production of this systematic review. In addition, they will use Excel (Microsoft Corp) spreadsheets to collect, organize, and document excluded and included abstracts and articles.

#### Selection Process

A 2-stage process will be used to select included guidelines and extract recommendations. The first stage involves 2 independent reviewers (NG-J and M-FP) reviewing the abstracts of papers that refer to guidelines to identify the guidelines that may be eligible. In the second stage, the same reviewers will evaluate the guidelines in the full articles. These guidelines will be independently reviewed to select those that meet the inclusion criteria. The reviewers will use hierarchical coding criteria to evaluate abstracts and full articles, as shown in Textbox 2. For each stage, they will resolve discrepancies by consensus and, if needed, a third reviewer (HF) will review the documents and contribute to a final consensus decision.

**Hierarchical coding criteria for abstracts and full articles.**

**Abstracts**

Hierarchical abstract coding (exclude if):

- The abstract is not in English
- The abstract clearly relates only an opinion, review, or commentary
- The abstract clearly does not involve a practice guideline or pinnacle practice information
- The abstract exclusively includes pediatric population
- The abstract does not relate to the acquired brain injury population
- The abstract clearly involves a duplicate publication to an accepted abstract

Otherwise, ACCEPT

**Full Articles**

Hierarchical full article coding (exclude if):

- The article is not in English
- The article clearly relates only an opinion, review, or commentary
- The article clearly does not involve a practice guideline or pinnacle practice information
- The guideline pertains to an adult sample (from a clinical context), even if there is mention of pediatric samples
- The guideline does not relate to the acquired brain injury population
- The guideline clearly involves overlap of information that is present in a more recent guideline

Otherwise, ACCEPT

#### Data Extraction, Appraisal of Guidelines and Recommendations, and Quality Assessment

Based on our objectives and research questions, data extraction will include the following information: (1) publication specifics (eg, country, organization, and date), (2) setting (eg, acute, rehabilitation, or community), (3) ABI population, (4) target health care professionals, and (5) categories of recommendations.

Two independent authors (LJ and NL) will first evaluate the selected clinical practice guidelines using the Appraisal of Guidelines for Research and Evaluation II (AGREE II) instrument, an international tool used to assess the quality and reporting of practice guidelines [38]. These authors will rate domains of methodological quality for each guideline according to AGREE II. When there is disagreement for a given rating, a third reviewer (TH) will also appraise the guideline with AGREE II.

Subsequently, reviewers NG-J and M-FP will independently evaluate guidelines containing oral care recommendations by using the National Health and Medical Research Council (NHMRC) instrument [39]. This tool allows the assessment and comparison of the levels of evidence and includes grades for the recommendations from the included guidelines. A third reviewer (HF) will resolve disagreements by appraising the recommendations in the same manner. Once the reviewers reach consensus, the 2 initial reviewers (NG-J and M-FP) will provide a descriptive synopsis of the levels of evidence for the oral care recommendations [39]. The risk of bias assessment within and across guidelines and recommendations is included in the Rigour of Development domain of AGREE II and the Evidence Base domain of the NHMRC. Because both tools include risk of bias elements in the quality appraisal, overall quality ratings for the guidelines and recommendations will reflect such information.

#### Strategy for Data Synthesis

We will use the AGREE II grades to synthesize the assessed guidelines data, and we will unify the levels of evidence and grades for oral care recommendations according to NHMRC levels to allow comparison and permit description of the evidence. We will retain all identified guidelines and describe their quality and level of evidence. A systematic narrative synthesis will be provided using textual description and tables to summarize and explain the scope, context, and consistency of the clinical practice guideline recommendations. The oral care recommendations will be compared across guidelines to identify similarities and discrepancies. Our systematic review will synthesize data from the guidelines based on quality rankings and levels of evidence for recommendations.

## Results

This project started in April 2019, when we developed the search strategy. The preliminary search of databases and websites yielded 863 and 787 citations, respectively, for a total of 1650. Data collection will start in August 2020 and we expect to begin disseminating the results in May 2021.

## Discussion

### Principal Findings

Even though good oral care prevents buccal and systemic infections after ABI [40], oral care recommendations are not always present in ABI guidelines [27-29]. The provision of oral care can reduce respiratory pathogens in saliva from dental plaque accumulation [41] and residual food or liquid in the oral cavity [42]. Research demonstrates that good oral hygiene decreases pneumonia rates [7] and thereby helps reduce mortality [9], morbidity, and length of hospitalization [6]. In stroke survivors, for instance, pneumonia is responsible for up to 28% of deaths [43].

However, achieving the means to provide good oral care to neurologically impaired survivors is complex due to physical [44-47], behavioral [48-50], emotional [11], and cognitive impairments [51-53]. Therefore, nursing staff need specific and attainable evidence-based recommendations. Currently, these professionals unfortunately often lack adequate training [14-17], sufficient time [20-22], or satisfactory directions [12,23,24] to provide high-quality oral care, especially in the context of dysphagia [54].

ABI guidelines worldwide endorse oral care for recovery and rehabilitation [27,51-56]. The prevention of pneumonia, notably in patients with dysphagia, is the main reason for recommending oral hygiene [27,40,55,57-59]. However, better functional outcomes, patient comfort, prevention of dental complications, good nutrition, and even prevention of sepsis are also related to mouth care after ABI [28,40,55,57].

### Expected Outcomes

The primary outcomes of this review will include methodological quality of guidelines and levels of evidence for oral care recommendations. We will apply priority rankings to the outcomes to inform health care professionals about highest-quality guidelines and recommendations for oral care, providing direction for future guidelines. The secondary outcome involves developing a best-practice protocol if the evidence is sufficient. There is a great need for a high-quality, process-oriented protocol for provision of oral care in neurologically impaired patients. Such a document could help orient health care professionals in their practice and provide direction for implementation research.

The results of this systematic review will be compared to the full guidelines, and differences between guidelines and recommendations will be explained. Findings may lead to the development of an oral care program that not only delivers safety within the hospital (prevention of complications) but helps clinicians identify opportunities for patients with ABI to independently manage their oral care, improving the long-term outcomes of this population.

### Conclusion

This systematic review will examine the methodological quality of existing recommendations for oral care of patients with ABI. Identifying and appraising the recommendations will support knowledge translation for evidence-based practice across the continuum of care. In addition, it will provide direction for improving future guidelines, developing clinical protocols, and addressing research gaps.

## Abbreviations

ABI: acquired brain injury
AGREE II: Appraisal of Guidelines for Research and Evaluation II
NHMRC: National Health and Medical Research Council
PRISMA: Preferred Reporting Items for Systematic Review and Meta-Analysis
